# Anticholinergic toxicity in a one-year-old male following ingestion of *Lupinus mutabilis* seeds: case report

**DOI:** 10.1590/1516-3180.2016.0157220517

**Published:** 2017-11-03

**Authors:** Adrian Ernesto Flores-Pamo, Elinor Pisano, Nilton Yhuri Carreazo

**Affiliations:** I MD. Resident Physician, School of Medicine, Universidad Nacional San Agustín de Arequipa (UNSA), Arequipa, Peru.; II MD. Resident Physician, Medstar Georgetown University Hospital, Washington, DC, United States.; III MD. Professor, School of Medicine, Universidad Peruana de Ciencias Aplicadas (UPC), Lima, Peru and Attending Physician, Pediatric Intensive Care Service, Hospital de Emergencias Pediátricas, Lima, Peru. orcid.org/0000-0002-5269-4855

**Keywords:** Lupinus, Foodborne diseases, Anticholinergic syndrome, Cholinergic antagonists, Alkaloids

## Abstract

**CONTEXT:**

The seeds from *Lupinus mutabilis* Sweet*,* also called “chocho”, are an important part of the diet in several countries in South America. Prior to consumption, processing is required to remove toxic alkaloids. These alkaloids are known to have pharmacological properties as antiarrhythmics, antimuscarinics and hypoglycemics.

**CASE REPORT:**

We report a case in which a one-year-old male initially presented with altered mental status and respiratory distress and subsequently developed symptoms of anticholinergic toxicity, after ingesting a large amount of chocho seeds.

**CONCLUSION:**

In spite of going through a difficult clinical condition, the subject evolved favorably through receiving supportive treatment. The seeds from *Lupinus mutabilis* provide nutritional benefits when consumed, but people need to know their risks when these seeds are consumed without proper preparation.

## INTRODUCTION

Ingestion of toxic substances is very common among young children. While household items such as medications are commonly ingested due to their similarity in appearance to candy, young patients can present with intoxication due to ingestion of plant matter as well. The majority of these occurrences are among patients less than six years of age.^1^ The true prevalence of intoxication due to ingestion of plant matter is difficult to determine, since many presentations are mild and may not be brought to medical attention.

Some of the most commonly known toxic plants are the deadly nightshade (*Atropa belladonna*) and jimson weed (*Datura stramonium*). These plants produce the anticholinergic alkaloids atropine, scopolamine and hyoscyamine. Yet these are not the only anticholinergic alkaloids present in plant matter: even the unripe buds or flowers of common garden plants, such as eggplants and tomatoes, can also cause an anticholinergic toxidrome if ingested.^2^

Supportive care is generally sufficient for treatment of anticholinergic toxicity. However, treatment with physostigmine may be indicated for severely affected patients.

## CASE REPORT

A one-year-old male child presented to the emergency room with acute onset of respiratory distress while sleeping, perioral cyanosis and severe cough. The patient’s mother said that she worked in a farmer’s market, on a stall where beans and vegetables were sold, and that she took her son to work with her every day. Among the harvested goods sold at the farmer’s center are unprocessed seeds from *Lupinus mutabilis* Sweet*,* known as “chocho”. The child had been seen placing a chocho seed in his mouth three hours before arrival at the hospital.

The physical examination upon presentation to the hospital was notable for stridor, which prompted bronchoscopic evaluation for a foreign body. However, no foreign body was found in the airway. Endoscopy revealed the presence of grains of chocho in the gastric cardia and stomach.

Six hours after initial presentation, the patient became irritable. He developed altered consciousness and shallow breathing. On examination, he demonstrated dilated, sluggishly reactive pupils and dry mucus membranes. His abdomen was distended and tympanic to percussion. His heart rate was 100/minute, respiratory rate 26/minute and oxygen saturation 98% on 30 FiO_2_ (fraction of inspired oxygen) through a nasal cannula. The arterial blood gas demonstrated pH 7.5, pCO_2_ 23, pO_2_ 135 and HCO_3_ 18.4. The blood parameters were: sodium 144 mEq/l, potassium 3.4 mEq/l, white blood cells (WBC) 8,800/mm^3^, hemoglobin 9.9 g%, platelets 316,000/mm^3^, glucose 107 mg/dl, creatinine 0.8 mg/dl and C-reactive protein (CRP) negative.

His altered mental status prompted admission to the intensive care unit (ICU) for monitoring and further evaluation. He was empirically started on intravenous fluids, ceftriaxone and mannitol. Computed tomography of the head was normal and lumbar puncture revealed normal cerebrospinal fluid; for this reason, all medications were discontinued and he was kept in the ICU for monitoring and supportive care.

After 24 hours, his mental status had improved and irritability diminished; his pupils returned to normal size and were reactive. On the following day, he developed liquid stools with elimination of the chocho seeds, and examinations demonstrated no remaining neurological signs. On the third day, he was discharged home.

## DISCUSSION

**Figure 1.**
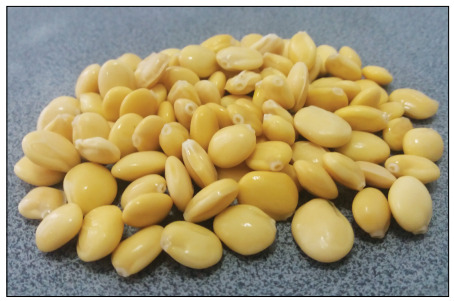
Typical appearance of processed chocho.

The Andean lupin, commonly known as “tarwi” or “chocho” (*Lupinus mutabilis* Sweet), is a legume native to the Andean region of South America, and is the only domesticated and cultivated species of the genus *Lupinus* (Figure 1). It is distributed from Colombia to the north of Argentina; with important presence in the agriculture of Ecuador, Peru and Bolivia.

Chocho is an important part of the native diet, with high protein and fat content.^3^ However, for consumption, the seed requires pre-treatment. This eliminates the toxic substances that it contains and which the plant uses for its natural defenses. This process is known as debittering (“desamargamiento”) and includes cleaning the harvested seeds of impurities (plant residues, soil and small stones), then soaking for a day, cooking in water for an hour, placing in an appropriate container (burlap sack or basket) and exposing to running water for 4-5 days (N).^4^

The alkaloids present in chocho are responsible for the bitter flavor and toxicity of this legume and can reach a content of 3.3% in unprepared seeds. The principal alkaloids present are lupinine, sparteine and hydroxylupinine. These compounds have been reported to have effects as central nervous system depressants, antiarrhythmics, hypoglycemics and antimuscarinics. The debittering process can reduce the concentration of these toxic substances to 0.003%.^5^ Given these innate toxic properties, it is common for farmers to use the seed without chemical pesticides.

The diagnosis of intoxication by *Lupinus mutabilis* is based on the clinical picture and antecedents of ingestion of chocho seeds. There have been previous case reports of symptoms after ingestion of the water that was used to process chocho, ingestion of the flour made of this legume and ingestion of the unprocessed seeds.^6^^,^^7^^,^^8^^,^^9^^,^^10^ The symptoms described are consistent with those found in cases of typical anticholinergic toxicity: dry mucus membranes, mydriasis, tachycardia, ileus, urinary retention and altered mental status (commonly remembered via the mnemonic “Dry as a bone, Blind as a bat, Mad as a hatter”).

This case is the first description of intoxication due to ingestion of the unprocessed beans by a pediatric patient. Even though the infant presented with the symptoms that have been described in this type of intoxication, the predominant clinical picture in this case was one of neurological symptoms, which may manifest as a range from mild disorientation to delirium and coma. In our patient, the alteration of consciousness directed the differential diagnosis towards possible meningitis or encephalitis. The treatment is supportive, with strict monitoring of vital signs; and the prognosis is favorable.^9^^,^^10^

We reviewed the literature in MEDLINE, Cochrane Library, LILACS and EMBASE using the keywords “Lupinus mutabilis” (Table 1). We found two related papers. A 1995 study (LILACS) reported that three adult patients in the city of Trujillo (Peru) became intoxicated due to consumption of chocho water (“*agua de chocho*”).^4^ The search in Medline and EMBASE located a second paper, published in 2012 (present in both databases): a 48-year-old woman who became intoxicated due to consumption of *agua de chocho*.^5^ We did not find any reports on pediatric cases.

**Table 1.** Search of the literature in medical databases for case reports on *Lupinus mutabilis*. The search was conducted on February 28, 2017

Database	Search strategies	Papers found	Related papers
PubMed	“Lupinus mutabilis”[All Fields] AND Case Reports[ptyp]	1	1
Cochrane Library	Lupinus mutabilis	1	0
LILACS	“lupinus mutabilis” AND (instance:”regional”) AND (db:(“LILACS”))	12	1
EMBASE	“Lupinus mutabilis”	30	1

Considering that the nutritional properties of chocho are continuing to be investigated (for example, its role as a hypoglycemic for treating diabetes mellitus), it can be expected that consumption of this product will increase.^11^^,^^12^ For this reason, it is important to inform consumers about the hazards of ingesting products containing *Lupinus* that have not been processed.

## CONCLUSIONS

In spite of going through a difficult clinical condition, the subject evolved favorably through receiving supportive treatment. The seeds from *Lupinus mutabilis* Sweet provide nutritional benefits when consumed, but people need to know their risks when these seeds are consumed without proper preparation.

## References

[1] Fine JS, McInerny TK, Adam HM, Campbell DE (2016). Poisoning. American Academy of Pediatrics Textbook of Pediatric Care.

[2] Carter K, Neuspiel DR (2010). Toxic plants. Pediatr Rev.

[3] Jacobsen SE, Mujica A (2008). Geographical distribution of the Andean lupin (Lupinus mutabilis Sweet). PGR Newsletter.

[4] Jacobsen SE, Mujica A (2006). El tarwi (Lupinus mutabilis Sweet.) y sus parientes silvestres. Botánica Económica de los Andes Centrales.

[5] Villacres E, Peralta E, Cuadrado L (2008). Propiedades y aplicaciones de los alcaloides del chocho (Lupinus mutabilis Sweet). INIAP ESPOCH SENACYT.

[6] Camacho Saavedra L, Uribe Uribe L (1995). Intoxicación por agua de Lupinus mutabilis (“Chocho”) [Lupinus mutabilis’s water intoxication]. Bol Soc Peru Med Interna.

[7] Ortega Duarte A, Martin-Sánchez FJ, Gonzales Castillo J, Ruiz Artacho P (2012). Intoxicación por “agua de chocho” [Lupinus mutabilis (chocho) water intoxication]. Medicina Clínica.

[8] Pingault NM, Gibbs RA, Barclay AM, Monaghan M (2009). Two cases of anticholinergic syndrome associated with consumption of bitter lupin flour. Med J Aust.

[9] Litkey J, Dailey MW (2007). Anticholinergic toxicity associated with the ingestion of lupini beans. Am J Emerg Med.

[10] Jamali S (2011). Dilated pupils, dry mouth and dizziness - a case study. Aust Fam Physician.

[11] Fornasini M, Castro J, Villacrés E (2012). Hypoglycemic effect of Lupinus mutabilis in healthy volunteers and subjects with dysglycemia. Nutr Hosp.

[12] Baldeón ME, Castro J, Villacrés E, Narváez L, Fornasini M (2012). Hypoglycemic effect of cooked Lupinus mutabilis and its purified alkaloids in subjects with type-2 diabetes. Nutr Hosp.

